# Swiss cost-effectiveness analysis of universal screening for Lynch syndrome of patients with colorectal cancer followed by cascade genetic testing of relatives

**DOI:** 10.1136/jmedgenet-2021-108062

**Published:** 2021-11-15

**Authors:** Islam Salikhanov, Karl Heinimann, Pierre Chappuis, Nicole Buerki, Rossella Graffeo, Viola Heinzelmann, Manuela Rabaglio, Monica Taborelli, Simon Wieser, Maria C. Katapodi

**Affiliations:** 1 Department of Clinical Research, University of Basel, Basel, Switzerland; 2 Institute for Medical Genetics and Pathology, University Hospital Basel, Basel, BS, Switzerland; 3 Oncogenetics Unit, Division of Oncology, Division of Genetic Medicine, Geneva University Hospital, Geneva, Switzerland; 4 Women's Clinic, Basel University Hospital, Basel, Switzerland; 5 Breast Unit of Southern Switzerland (CSSI), Oncology Institute of Southern Switzerland, Bellinzona, Switzerland; 6 Medical Oncology, Inselspital University Hospital Bern, Bern, Switzerland; 7 Genetic Services, Oncology Institute of Southern Switzerland, Bellinzona, Switzerland; 8 Winterthur Institute of Health Economics, Zurich University of Applied Sciences, Winterthur, Switzerland

**Keywords:** costs and cost analysis, gastrointestinal diseases, genetic counseling, health care economics and organizations, genetic testing

## Abstract

**Background:**

We estimated the cost-effectiveness of universal DNA screening for Lynch syndrome (LS) among newly diagnosed patients with colorectal cancer (CRC) followed by cascade screening of relatives from the Swiss healthcare system perspective.

**Methods:**

We integrated decision trees with Markov models to calculate incremental cost per quality-adjusted life-year saved by screening all patients with CRC (alternative strategy) compared with CRC tumour-based testing followed by DNA sequencing (current strategy).

**Results:**

The alternative strategy has an incremental cost-effectiveness ratio of CHF65 058 compared with the current strategy, which is cost-effective according to Swiss standards. Based on annual incidence of CRC in Switzerland, universal DNA screening correctly identifies all 123 patients with CRC with LS, prevents 17 LS deaths and avoids 19 CRC cases, while the current strategy leads to 32 false negative results and 253 LS cases lost to follow-up. One way and probabilistic sensitivity analyses showed that universal DNA testing is cost-effective in around 80% of scenarios, and that the cost of DNA testing and the number of invited relatives per LS case determine the cost-effectiveness ratio.

**Conclusion:**

Results can inform policymakers, healthcare providers and insurance companies about the costs and benefits associated with universal screening for LS and cascade genetic testing of relatives.

## Introduction

Lynch syndrome (LS) is a cancer predisposition syndrome that confers a 12%–52% lifetime risk for colorectal cancer (CRC), and a 13%–60% lifetime risk for endometrial cancer, while the corresponding risks in the general population are 5%–6% and 2%–3%, respectively.^1^ LS is also associated with glioblastomas and with gastric, ovarian, small bowel, pancreatic and urothelial cancers.^2^ Germline pathogenic variants in mismatch repair (*MMR*) genes (ie, *MLH1, MSH2, MSH6* or *PMS2*) or deletions in the 3’end of the *EPCAM* gene predispose to LS.^3^ Pathogenic variants are inherited in an autosomal-dominant manner; for every LS case, there are multiple blood relatives with the same pathogenic variant. First-degree and second-degree relatives (FDR, SDR) have 50% and 25% probability, respectively, of inheriting the pathogenic variant.^4^

LS is a common cancer predisposition condition with an estimated population frequency 1:279.^5^ However, LS remains largely undetected due to different associated cancer types and the lack of clear diagnostic criteria. The Amsterdam II and revised Bethesda guidelines that have been traditionally used to identify individuals at risk for LS can miss 23%–50% of cases.^6–8^ Moreover, LS often occurs before screening recommendations apply, resulting in late identification of cases.^9^ Due to these limitations, only a fraction of LS cases is referred for genetic evaluation and less than 10% receive genetic testing.^10^ Underdiagnosis of LS results in a significant number of patients and blood relatives not receiving appropriate care and in unnecessary and preventable morbidity and mortality.^11^ LS cases with CRC can benefit from treatment with monoclonal antibodies or immune check-point inhibitors in combination with adjuvant chemotherapy.^12^ Colonoscopy decreases CRC morbidity and mortality by detecting the disease at earlier stages and can also be preventive by allowing for endoscopic removal of preneoplastic lesions/polyps.^13 14^ Additional prevention and screening methods, such as daily aspirin, upper gastrointestinal endoscopy and prophylactic surgery can be tailored to individual needs of LS cases.^15^

This study presents a Swiss-based cost-effectiveness analysis of genetic screening for LS provided to all newly diagnosed patients with CRC, followed by cascade genetic screening of blood relatives. Although LS cases may present with different forms of cancer, we focus on CRC because it is the most common cancer associated with the syndrome. Screening for LS among all newly diagnosed CRC cases, irrespective of age and family history, followed by cascade testing of blood relatives, has been embraced by the Evaluation of Genomic Applications in Practice and Prevention (EGAPP) Working Group.^16^ Blood relatives of LS cases can be tested inexpensively and with 100% accuracy, and those who test negative are excluded from early screening and preventive interventions.^17^ Diagnosis of LS with this protocol has 85% sensitivity and 90% specificity.^10 18^

We compared two strategies: the current strategy implemented in Switzerland involves preliminary tumour testing with immunohistochemistry (IHC), *BRAF V600E* and germline DNA sequencing of a fraction of patients with CRC and inviting four FDR and/or SDR per every identified LS case for cascade testing. The alternative strategy involves DNA sequencing of all newly diagnosed patients with CRC, and inviting four FDR and/or SDR per every identified LS case for cascade testing. The alternative strategy does not include preliminary tumour testing but focuses on germline genetic testing of patients with CRC followed by cascade testing of relatives. The study examines whether the alternative strategy for identifying LS cases is economically reasonable, considering the perspective of the Swiss healthcare system.

## Materials and methods

We developed an analytic model combining decision trees with Markov modelling to estimate the cost-effectiveness of universal screening for LS for all newly diagnosed patients with CRC followed by cascade testing. We estimated costs of tumour testing, DNA sequencing, colonoscopy and treatment. Decision trees represent the structure of LS screening, modelling possible decisions and outcomes and displaying the algorithm behind the processes leading to genetic testing. The integration of Markov modelling helped conduct probabilistic forecasting and predictive modelling of future events to calculate costs and outcomes (number of CRC cases, deaths, CRC avoided) associated with genetic testing over the period of 50 years. Each Markov model is associated with a corresponding end node of the decision tree. To find incremental cost-effectiveness ratio (ICER), we calculated differences in total costs and number of gained quality-adjusted life years (QALYs) for each strategy (table 1).

**Table 1:** Key design criteria of the analysis

Population	Individuals newly diagnosed with CRC and FDR and SDR
Intervention	DNA sequencing of all newly diagnosed CRC cases and cascade genetic testing of four or more FDR and SDR of identified LS cases
Comparator	Current strategy with IHC, *BRAF V600E* and DNA sequencing for a proportion of newly diagnosed CRC cases and cascade testing of four FDR and/or SDR
Outcome	QALYs saved
Model type	Decision trees integrated with Markov models
Time horizon	Lifetime/50 years
Perspective	Swiss healthcare system
Costs	Swiss francs (CHF)
Discounting	3% per year
Cost-effectiveness threshold	CHF100000 per QALY

CRC, colorectal cancer; FDR, first-degree relative; IHC, immunohistochemistry; LS, Lynch syndrome; QALYs, quality-adjusted life years; SDR, second-degree relative.

### Model inputs

Data on costs have been obtained from the University Hospital Basel, the Geneva University Hospitals and published literature.^10^ Costs applied in modelling include detection of LS cases among patients with CRC and relatives; colonoscopy; CRC treatment. Costs associated with detection of LS cases among CRC incident cases include genetic consultations; tumour-based testing (IHC for the four *MMR* proteins and *BRAF* V600E) and germline screening. In Switzerland, germline screening for LS diagnosis is conducted in two steps: sequencing of two to four *MMR* genes by next generation sequencing (NGS); Sanger sequencing of selected exons and gene dosage analysis by multiplex ligation-dependent probe amplification conducted to confirm NGS findings. Costs of LS screening for relatives include genetic consultations and carrier testing for the identified pathogenic variant (cascade testing). All costs applied in modelling are provided in the supplementary materials and are expressed in 2020 Swiss Francs (CHF).

Parameters used in decision trees and Markov modelling are based on an EGAPP review.^16^ All financial, epidemiological, and clinical model inputs, including sensitivity and specificity of IHC, *BRAF* V600E and DNA sequencing are literature-based^10^ (see online supplemental materials). For IHC and *BRAF* V600E, we applied sensitivity of 83.0% and 69.0%, respectively, and specificity of 88.8% and 99.0% respectively. We assumed 99.5% sensitivity and 99.9% specificity of DNA sequencing. We also assumed that 79% of relatives testing positive for LS would accept increased surveillance. Transition probabilities between states (Healthy, CRC, mCRC, Death) and stage distributions of CRC in screened and unscreened populations are literature-based and were used to calculate costs of treatment of the corresponding CRC stage.^10 18^ Risks associated with colonoscopy, such as perforation, bleeding and death, have also been incorporated into the model.^19 20^ To separate CRC-related deaths and deaths from other causes, we used annual crude death rate in Switzerland equal to 0 0079 in both strategies.^21^ Based on current Swiss practices, we assume that identified LS cases will invite four CRC-free FDR and/or SDR for cascade testing. Among relatives who agree to genetic testing, those who test positive for LS are offered biennial colonoscopy, starting at 25 years old.^22^ The probability of identifying the familial pathogenic variant among FDR is 45% and decreases to around 25% in SDR. The probability to identify LS cases with cascade testing in both FDR and SDR is around 35%.^10^ Both strategies were ranked according to costs in CHF and effects in QALYs. We calculated ICER of net costs per QALYs saved. The cost-effectiveness analysis was conducted based on the Consolidated Health Economic Evaluation Reporting Standards statement.^23^

10.1136/jmedgenet-2021-108062.supp1 Supplementary data

### Decision trees

**Figure 1:**
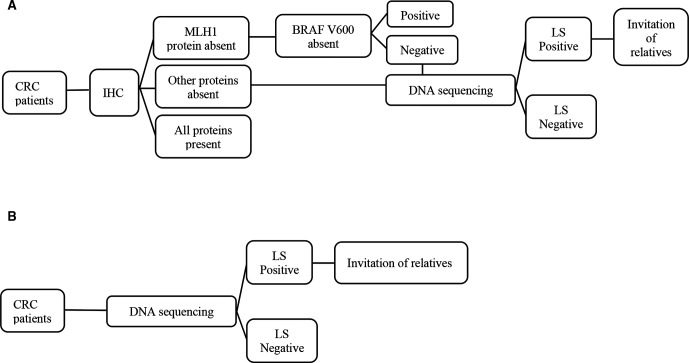
Schematic representation of compared testing strategies for LS for patients newly diagnosed with CRC. (A) Strategy 1 represents current screening for LS including two tumour analyses (IHC and BRAF V600), followed by DNA sequencing for suspicious cases. (B) Strategy 2 represents alternative universal DNA sequencing for all CRC cases followed by cascade genetic testing of relatives of mutation carriers. CRC, colorectal cancer; IHC, immunohistochemistry; LS, Lynch syndrome.

Decision trees represent the detailed structure of events and outcomes associated with each of the two strategies based on epidemiological data and Swiss clinical parameters for CRC and LS (see online supplemental materials). For the current strategy, we calculated the number of false negative and false positive results associated with IHC and *BRAF* V600 testing and we assessed the number of patients with CRC lost-to-follow up. For the alternative strategy, the decision tree allowed us to calculate the number of patients with CRC and relatives with pathogenic variants in *MMR* genes and to evaluate the number of individuals who develop CRC (figure 1).

The current strategy consists of three phases: identifying carriers of pathogenic variants in *MMR* genes among newly diagnosed patients with CRC by screening tumour tissue with IHC, *BRAF* V600E and DNA sequencing; offering carrier testing to FDR and/or SDR of identified LS cases; using colonoscopy for early detection of CRC among relatives with LS. IHC is conducted routinely in Switzerland and patients with loss of *MLH1* expression undergo testing for *BRAF* V600E, while patients whose tumours demonstrate loss of *MSH2, MSH6* or *PMS2* expression undergo germline DNA sequencing directly after IHC. Cases identified with LS receive recommendations to notify their blood relatives for cascade testing. The cost of carrier testing for relatives is substantially lower because the location of the pathogenic variant is known (ie, CHF3500 for patients with CRC vs CHF400 for relatives). This cost is covered only for FDR in the Swiss healthcare system. The alternative strategy assumes universal germline testing for all newly-diagnosed CRC cases followed by cascade testing of four FDR and/or SDR of LS cases. The alternative strategy has high sensitivity and specificity to detect LS and is consistent with evidence-based recommendations for cascade screening for ‘actionable’ hereditary cancer syndromes.^4 18^

### Markov modelling

Markov modelling estimated the long-term costs and the number of annually gained QALYs in both strategies. Markov models were based on four states: healthy, CRC, metachronous CRC (mCRC) and death. Once a person is diagnosed with CRC, the disease could progress to mCRC or death. Markov models used 1 year cycle length and were continued for 50 years, assuming that all cohort participants will be dead by the end of this time frame. We modelled annual transition probability from CRC to mCRC of around 1%, based on the risk of mCRC depending on the affected *MMR* gene and considering a time frame ranging from 6 months to 12 years post initial CRC diagnosis.^24–26^ We modelled risk of developing cancer (mCRC) among screened and unscreened populations according to evidence of frequency of colonoscopy and its CRC/mCRC risk reduction.^13 26^ Modelling assumptions are conservative, therefore, intentionally made unfavourable for the cost-effectiveness of the alternative strategy. Analyses were performed using Microsoft Excel 2016 (Microsoft) with a discount rate of 3% for both costs and effects (see online supplemental materials).

### Sensitivity analysis

A deterministic one-way sensitivity analysis estimated the effects of variations in each input parameter on overall cost-effectiveness of the alternative strategy. Probabilistic sensitivity analysis tested the robustness of our modelling and assessed the overall likelihood of the alternative strategy to be not cost-effective. Probabilistic sensitivity analysis tested 1000 scenarios varying different parameters, such as number of CRC cases accepting germline genetic testing, number of relatives accepting carrier testing, probability to develop cancer among relatives positive for LS, compliance with colonoscopy and lost-to-follow up rate.

## Results

### Decision trees

Modelling of the two LS screening strategies begins with a cohort of 4100 newly diagnosed patients with CRC, based on the annual number of CRC incident cases in Switzerland.^27^ Assuming that 3% of all newly diagnosed patients with CRC are affected by LS, there are 123 LS cases with CRC.^28^ With the current strategy, all 4100 newly diagnosed patients with CRC are offered tumour-based testing. Among them, 32 cases have false negative results, while 33 patients with CRC are correctly identified with LS, counselled and invite relatives for cascade testing. With the current strategy, 253 patients with CRC are lost to follow-up before DNA sequencing. Using a conservative approach, we assumed that only 50% of relatives accept cascade testing, while only 79% of relatives identified with LS undergo biennial colonoscopy. With the alternative strategy, all 123 of 4100 newly diagnosed patients with CRC are identified as LS cases, and 492 relatives are invited for cascade testing (see online supplemental materials). CRC stages are classified according to the Duke’s Classification.^29^

### Markov modelling

**Figure 2:**
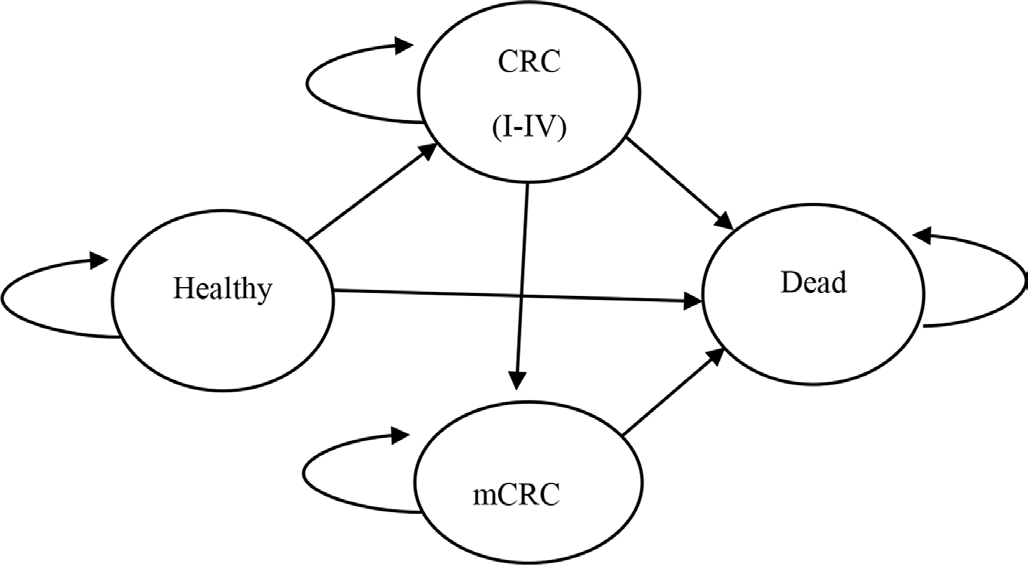
Markov model with the modelled transition probabilities between health states: healthy, CRC, metachronous CRC and death. CRC, colorectal cancer.

Figure 2 shows the Markov models with transitions between the four disease states. The Markov models account for costs associated with colonoscopy and CRC treatment over 50 years. Each cancer-free relative diagnosed with LS is recommended to undergo biennial colonoscopy, starting at the age of 25. We assumed that colonoscopy decreases the lifetime risk of CRC by 67%; therefore, more LS cases tend to stay healthy during each following year compared with those who do not undergo colonoscopy. While colonoscopy is associated with higher costs of surveillance, it reduces overall treatment costs, due to early detection of CRC and mCRC while also having a favourable effect on stage distribution of CRC. Swiss anecdotal data suggest that a proportion of relatives who refuse cascade testing may still elect to have a colonoscopy every 3 years. We assumed that this frequency of colonoscopy also decreases the risk of CRC but only by 25%. Relatives testing negative for LS are assumed to have a population-level risk of CRC; therefore, they are offered colonoscopy screening every 10 years, starting at the age of 50 (see online supplemental materials).

Total screening costs for patients with CRC and relatives ranged between CHF136 966 947 with the current strategy and CHF150 691 700 with the alternative strategy. ICER of universal LS screening with cascade testing of four relatives is CHF65 058 per QALY gained. This is cost-effective in Swiss healthcare settings, where the cost-effectiveness threshold is assumed to be CHF100 000 per QALY saved. The expected discounted gained QALYs ranged between CHF361 147 with the current strategy and CHF361 358 in the alternative strategy. Universal LS screening gained 211 more QALYs at the additional cost of CHF13 724 753 compared with the current strategy (table 2). LS is associated with high rates of secondary CRC; therefore, mCRC was incorporated into our model to calculate associated costs and mortality. According to our model, the alternative strategy prevents 17 deaths at the cost of CHF785 645 per death avoided. Moreover, the alternative strategy avoids 18 cases of CRC and one case of mCRC, compared with the current strategy (table 3).

**Table 2:** Costs associated with genetic testing for LS

Costs for cohort (CHF)	Current strategy	Alternative strategy
IHC	1 476 000	0
*BRAF* V600E	50 809	0
DNA sequencing	497 808	14 350 000
DNA sequencing for relatives	29 442	110 700
Colonoscopy and treatment	134 912 887	136 231 000
Total	136 966 947	150 691 700
QALYs gained	361 147	361 358
Cost difference	13 724 753	
QALYs difference	211	
ICER	65 058	

ICER, incremental cost-effectiveness ratio; IHC, immunohistochemistry; LS, Lynch syndrome; QALYs, quality-adjusted life years.

**Table 3:** Health outcomes associated with compared screening strategies

Health outcome (N)	Current strategy	Alternative strategy	Difference
QALYs gained	361 147	361 358	211
Relatives with CRC	814	795	−18
Relatives with mCRC	33	32	−1
Deaths	5612	5595	−17
Patients with CRC with LS identified	33	123	90
Relatives with LS identified	29	111	81

CRC, colorectal cancer; LS, Lynch syndrome; QALYs, quality-adjusted life years.

### Sensitivity analyses

**Figure 3:**
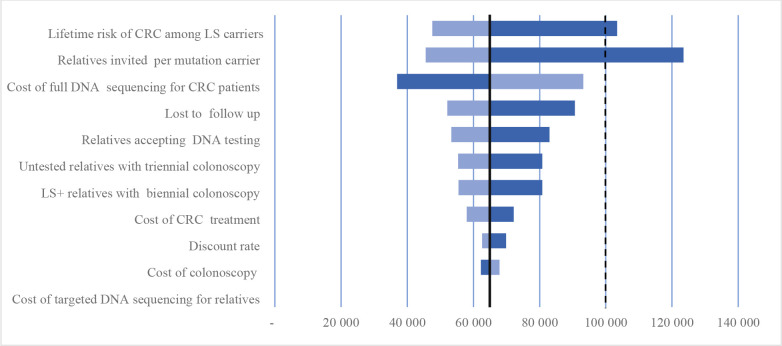
Tornado diagram. One-way sensitivity analysis for universal DNA sequencing (Strategy 2). The Y axis shows tested parameters and the X axis shows cost per QALY saved. The change in cost-effectiveness associated with 50% decrease in each parameter is depicted by the darker bars, which indicate higher cost per QALY saved (ie, less cost-effective), the change associated with 50% increase in each parameter is depicted by the lighter bars, which indicate lower cost per QALY saved (ie, more cost-effective). The solid vertical line represents the default cost-effectiveness ratio of CHF 65 058 per QALY saved. The dotted vertical lines indicate cost-effectiveness threshold of CHF 100 000 in Switzerland. CRC, colorectal cancer; LS, Lynch syndrome; QALY, quality-adjusted life years.

Figure 3 shows results of one-way sensitivity analysis using 11 parameters in descending importance. This analysis revealed that the number of invited relatives and cost of germline DNA sequencing for patients with CRC had a major impact on the outcome. Decreasing the cost of DNA sequencing from CHF3500 to CHF2500 reduces the ICER of the alternative strategy to CHF49 947. Costs of tumour tests and carrier testing for relatives did not have a substantial effect on the overall cost-effectiveness of the alternative strategy. In cases when only two relatives per LS case are invited for cascade testing, the alternative strategy costs CHF123 483 per QALY and is not cost-effective. Reducing the risk of CRC in relatives diagnosed with LS to 25% increases the cost of the programme to CHF103 385 per QALY, exceeding the cost-effectiveness threshold.

Probabilistic sensitivity analysis was conducted using five parameters, that is, patients with CRC lost-to-follow up, number of invited relatives per LS case, proportion of tested relatives and compliance with biennial colonoscopy. Results of probabilistic sensitivity analysis suggest that the number of invited relatives per LS case is the most influential factor affecting the overall cost-effectiveness of the alternative strategy. In a Monte-Carlo simulation of 5000 scenarios, the ICER of the alternative strategy varied between CHF33 075 and CHF316 010, with a mean of CHF73 792. Increasing the number of invited relatives from four to seven decreases the ICER from CHF65 058 to about CHF45 000. Given 50% acceptance rate among relatives, increasing the number of invited relatives by one per LS case decreases the ICER on average by CHF5000. Including SDR reduces the ICER from CHF65 058 to CHF32 886. Finally, increasing the frequency of colonoscopy from biennial to annual increases the ICER from CHF65 058 to CHF70 536 per QALY gained. The universal strategy has a probability of 80% to be cost-effective given the threshold of CHF100 000 per QALY. We assumed normal distribution in epidemiological and clinical parameters and gamma distribution in costs.

## Discussion

We estimated the cost-effectiveness of universal screening for LS among all newly diagnosed patients with CRC with cascade testing of relatives as a measure of identifying LS cases. Our cost-effectiveness analysis incorporated costs of carrier testing of relatives, an aspect that is often omitted from cost-effectiveness analyses of genetic testing technologies.^30^ Universal LS screening with cascade testing of relatives is cost-effective in around 80% of scenarios. The alternative strategy prevents 17 deaths at the cost of CHF785 645 per death avoided, with ICER of CHF65 058 per QALY saved and below the acceptable Swiss cost-effectiveness threshold of CHF100 000. Since the risk of death from other causes was equal in both strategies, the observed difference of 17 deaths is associated with CRC. The number of prevented deaths is associated with annual transitions from Healthy to CRC, and from CRC to death, which are substantially lower in tested and screened populations.^13^

The current strategy for LS screening in Switzerland involves two tumour-based tests (IHC and *BRAF V600*) followed by DNA sequencing. It is possible that anticipating results from three sequential tests, including tumour biopsy, causes discomfort to patients and their families, resulting in 64% lost-to-follow-up rate among patients with CRC.^31^ Germline DNA screening has almost 100% validity and, therefore, identifies all LS cases and eliminates false negative and false positive diagnoses. This may result in modification of CRC treatment with better patient outcomes.^12^ It also prevents patients with CRC from being lost-to-follow-up and deaths of undiagnosed LS cases and identifies a higher number of cancer-free relatives and new CRC cases at early stages through colonoscopy screening. The advantages of universal LS screening are to simplify the process of identifying LS cases, possibly provide personalised treatment to those with CRC and to identify cancer-free relatives with LS who can benefit from colonoscopy screening starting at a younger age. Despite wide application of the Bethesda and Amsterdam guidelines for initiating LS screening, they are not routinely applied in Switzerland because they can miss up to 50% of LS cases due to poor data quality, small family size or lack of awareness of cancer cases in the family.^6^ Implementing these criteria is also associated with more costs and burden of data collection, interpretation and quality assurance.

Despite the significant cost associated with offering DNA testing to all patients with CRC, universal LS screening improves the overall utility of genetic testing at a reasonable cost. Probabilistic sensitivity analysis revealed that universal LS screening is cost-effective in around 80% of scenarios, while one-way sensitivity analysis showed that it is cost-effective in the majority of possible scenarios. Our model was limited to LS and germline sequencing to either two or four *MMR* genes, while NGS-panel testing is highly advisable for all CRC cases because it can identify several other cancer syndromes.^32^ Identification of non-LS-associated CRC cases would likely increase the cost-effectiveness of universal LS screening as more families with inherited predisposition to cancer would be identified and provided with risk reducing strategies.

One scenario that was unfavourable for universal LS screening was to decrease the number of invited relatives to two per LS case. This scenario increases the ICER to about CHF123 000, which is unfavourable for the Swiss healthcare system. Although our models assumed testing of four relatives per LS case, Swiss data demonstrate that four LS cases invited more than 50 relatives for cascade testing.^31^ Implementing strategies to facilitate cascade testing, for example, mailing of saliva kits and family-based telephone or web-based counselling, holds promise to enhance the cost-effectiveness of universal LS screening.^33 34^ Expanding insurance coverage to SDR, who are currently not covered for carrier testing in the Swiss healthcare system, will increase identification of an underdiagnosed syndrome at the population level and significantly reduce the time needed to detect LS predisposing variants.^35^ Moreover, although the cost of targeted testing is significantly lower (CHF400 vs CHF3500), lack of insurance coverage for cascade testing may be a significant barrier accessing specialised genetic services for segments of the population and further contributes to healthcare disparities.^36^

Another unfavourable scenario for universal LS screening was to reduce the risk of CRC among relatives diagnosed with LS to 25%, which increased the cost of the programme to CHF103 385 per QALY above the cost-effectiveness threshold. Cumulative risks of LS-associated cancers depend on sex and distribution of *MMR* gene pathogenic variants.^2^ *MLH1* and *MSH2* pathogenic variants raise the lifetime risk of CRC to around 50%,^37^ while *PMS2* variants raise the lifetime risk of CRC up to 12% and 13% for endometrial cancer.^2 32^ Given that *PMS2* pathogenic variants are less frequent, we consider that a 40% CRC risk among relatives identified with LS through cascade testing is a realistic assumption, consistent with the German-based model (42% risk of CRC among LS cases by age 80 years) and with EGAAPP consensus.^5 18 20^

Finally, the cost of carrier testing for relatives and the cost of colonoscopy do not have substantial effects on ICER. Our model assumed 79% compliance with biannual colonoscopy.^10^ Other studies reported colonoscopy compliance ranging from 67% to 97%.^20 38 39^ Using one-way sensitivity analysis and probabilistic sensitivity analysis, we varied compliance rates between 58% and 100%. Decreasing colonoscopy compliance to 58% increased the cost-effectiveness ratio to almost CHF 81 000 which is still below the cost-effectiveness threshold.

Our findings and parameters are compatible with cost-effectiveness analyses of LS screening conducted in the USA and in Australia. The US-based model demonstrated the cost-effectiveness of a strategy including IHC, *BRAF* and DNA sequencing with cascade testing of 12 relatives per LS case and ICER of $50 000 per life-year saved.^10^ Based on a second US-based model, using a predictive model to stratify individuals into different levels of cancer risk, followed by IHC and germline DNA testing resulted in ICER of $35 143 per life-year saved with the age cut-off at 25–35 years.^40^ The Australian-based model showed a cost-effectiveness ratio of $61 235 per life-year saved with annual colonoscopy and with no age limit.^19^ Our findings are conflicting with a German-based model, reporting that the ICER of universal LS screening is €4 188 036, and it is not cost-effective compared with tumour-based screening.^18^ This substantial difference in costs between the German and our model might be explained by several reasons. The German model assumed higher costs of DNA sequencing, lower number of tested relatives per LS case, and lower proportion of cancer-free relatives participating in colonoscopy screening. In our model, one-way sensitivity analysis revealed that the number of tested relatives per LS case and the cost of germline DNA testing had the highest impact on overall cost-effectiveness ratio. In most studies, the number of tested relatives per LS case had the highest impact on results, despite substantial differences in input parameters.

The first limitation of our study is that we evaluated the cost-effectiveness of universal LS screening based solely on patients with CRC, while LS is also associated with other types of cancer, including endometrial cancer. We focused on CRC because it is commonly associated with the syndrome and affects both women and men. Including screening for endometrial cancer will probably increase the cost-effectiveness of universal LS screening, given strategies for early prevention and risk-reducing surgery.^41^ International groups further emphasise the importance of genetic testing of women with endometrial cancer for early identification of healthy LS cases.^42^ We also assumed that 50% of relatives who refused cascade testing might undergo frequent colonoscopy screening. The exact proportion of relatives undergoing frequent colonoscopies is unknown and our analyses may not be accurate regarding the potential benefits of universal LS screening. Our model did not account for the proportion of CRC cases identified with variants of unknown significance (VUS). VUS rate is reportedly 6% for *MLH1/MSH2/MSH6/EPCAM* genes and 4% for *PMS2*,^43^ while Swiss lab experience indicates 10% frequency of VUS. Although these cases will not yield downstream cascade testing, they are managed with increased frequency of colonoscopy,^44^ which may yield changes in surveillance among relatives. Nevertheless, we acknowledge that VUS cases will likely decrease the cost-effectiveness of universal genetic screening in real-world settings. Finally, in real-world settings, it is unlikely to obtain consent for germline genetic testing from 100% of CRC cases, which may have an effect on cost-effectiveness of universal LS screening. A Swiss-based single-centre study reported approximately 14% refusal rate of germline testing among patients with CRC.^31^ Our one-way sensitivity analyses showed that when only 50% of patients with new CRC accept germline testing, the overall cost of the alternative strategy increases to almost CHF78 000 per QALY saved, still below the cost-effectiveness threshold.

We demonstrate that universal LS screening with cascade testing of relatives results in substantial benefits for the Swiss healthcare system at a reasonable cost. Our findings provide evidence needed to inform policymakers, healthcare providers and insurance companies about the costs and health benefits associated with universal LS screening and cascade testing of relatives as a public health intervention, supporting NICE guidelines.^45^ The overall cost-effectiveness of this approach depends on the costs of DNA sequencing and the willingness of patients and relatives to be tested. The cost of DNA sequencing depends on how many genetic variations are analysed; during the past 15 years, this cost has dramatically decreased, and it is foreseen that this trend will continue.^46^ Close coordination of different stakeholders, such as primary care providers, specialists, genetic clinicians and laboratories is crucial to encourage and educate the public about the importance of screening for LS. Further research needs to examine the cost-benefit ratio of universal LS screening, since individual preferences for genetic testing should be elicited and used in shared decision-making.^47^

## Data Availability

All data relevant to the study are included in the article or uploaded as supplementary information.
