# Supplementary material for: Swiss cost-effectiveness analysis of universal screening for Lynch syndrome of patients with colorectal cancer followed by cascade genetic testing of relatives
Source: J Med Genet. 2021 Nov 15;59(9):924–30. doi: 10.1136/jmedgenet-2021-108062 (PMC9411888; doi:10.1136/jmedgenet-2021-108062)
Supplement: Supplementary data [file jmedgenet-2021-108062supp001.pdf]

Supplementary material 1

A Swiss cost-effectiveness analysis of universal screening for Lynch syndrome of colorectal cancer patients followed by cascade genetic testing of relatives.

Journal of Medical Genetics

Islam Salikhanov MD, MPH. University of Basel, Department of Clinical Research. Basel, Switzerland

Karl Heinimann Dr. med. Dr. phil. University Hospital Basel. Department of Biomedicine. Basel, Switzerland

Pierre O. Chappuis Dr. med. University Hospitals of Geneva. Division of Oncology and Division of Genetic Medicine. Geneva, Switzerland

Nicole Bürki PD med. University Hospital Basel. Basel, Switzerland

Rossella Graffeo. Department of medical oncology, Inselspital, Bern University Hospital, University of Bern, Switzerland

Viola Heinzelmann-Schwarz Dr. med. University Hospital Basel. Basel, Switzerland

Manuela Rabaglio Dr. med. University Hospital of Bern. Bern, Switzerland

Monica Taborelli Dr.Biol. Oncology Institute of Southern Switzerland. Bellinzona, Switzerland

Simon Wieser PhD#. Zurich University of Applied Sciences. Zurich, Switzerland

Maria Katapodi PhD #* for the CASCADE Consortium. University of Basel, Department of Clinical Research. Email: maria.katapodi@unibas.ch Basel, Switzerland ORCID:0000-0003-3903-3750

Keywords: cost-effectiveness, genetic testing, Lynch syndrome, colorectal cancer, CASCADE consortium

#Equal Contribution

* Corresponding Author

Table 1 Model input parameters

Variable		Confidence interval	Source
**Validity of genetic tests**			
Number of patients with CRC	4100		5
Prevalence of LS among newly diagnosed patients with CRC	3%		10
IHC sensitivity	83%	63-96%	10
IHC specificity	88.8%	83-94%	10
BRAF sensitivity	69%	50-85%	10
BRAF specificity	99%	98-99%	10
DNA sensitivity	99%	98-99%	3
DNA specificity	100%		4
**Stage distribution of CRC at diagnosis without colonoscopy**			
Localized—Stage 1	40%		10
Regional—Stage 2	36%		10
Distant—Stage 3	19%		10
Unstaged—Stage 4	5%		10
**Stage distribution of CRC at diagnosis with colonoscopy**			
Localized—Stage 1	77%		10
Regional—Stage 2	15%		10
Distant—Stage 3	6%		10
Unstaged—Stage 4	2%		10
**Costs**			
IHC for the DNA MMR proteins	CHF 360		University Hospital of Basel
BRAF V600E	CHF 290		University Hospital of Basel
DNA sequencing for patients	CHF 3500		University Hospital of Basel
DNA sequencing for relatives	CHF 376		University Hospital of Basel
Cost of colonoscopy	CHF 1000		University Hospital of Basel
**Costs of treatment**			
Stage I	CHF 25,516		10
Stage II	CHF 28,166		10
Stage III	CHF 31,907		10
Stage IV	CHF 45,393		1
Weighted cost of risks after colonoscopy	CHF 27		10
**Epidemiological variables**			
Number of invited relatives per CRC patient	4	1-7	10

Proportion of relatives who take DNA test	50%		10
LS prevalence among relatives	45%		10
Decrease in CRC risk after biennial colonoscopy	67%	38-83%	3; 7
Decrease in CRC risk after colonoscopy every 3 years	30%	20-40%	Assumption
CRC risk in probands with LS	40%	27-54%	10
CRC risk in probands without LS	5,5%	4.5-6.5%	2
Proportion of MLH1 loss of expression	32%	18-46%	10
Proportion of standard colonoscopy vs. colonoscopy every 3 years	50%	30-70%	Assumption
Proportion of *MLH1* germline mutations among IHC positives	32%		10
**Quality of life**			
Healthy	1		Assumption
CRC	0,77		6
mCRC (Advanced cancer)	0,5		6
Dead	0		Assumption

1 Daly MB, Pilarski R, Yurgelun MB, Berry MP, Buys SS, Dickson P, Domchek SM, Elkhanany A, Friedman S, Garber JE, Goggins M, Hutton ML, Khan S, Klein C, Kohlmann W, Kurian AW, Laronga C, Litton JK, Mak JS, Menendez CS, Merajver SD, Norquist BS, Offit K, Pal T, Pederson HJ, Reiser G, Shannon KM, Visvanathan K, Weitzel JN, Wick MJ, Wisinski KB, Dwyer MA, Darlow SD. NCCN Guidelines Insights: Genetic/Familial High-Risk Assessment: Breast, Ovarian, and Pancreatic, Version 1.2020. J Natl Compr Canc Netw 2020;18. doi:10.6004/jnccn.2020.0017

2 Lynch HT, Snyder CL, Shaw TG, Heinen CD, Hitchins MP. Milestones of Lynch syndrome: 1895–2015. Nat Rev Cancer 2015;15. doi:10.1038/nrc3878

3 Snowsill T, Huxley N, Hoyle M, Jones-Hughes T, Coelho H, Cooper C, Frayling I, Hyde C. A systematic review and economic evaluation of diagnostic strategies for Lynch syndrome. Health Technol Assess 2014;18. doi:10.3310/hta18580

4 Severin F, Stollenwerk B, Holinski-Feder E, Meyer E, Heinemann V, Giessen-Jung C, Rogowski W. Economic evaluation of genetic screening for Lynch syndrome in Germany. Genet Med 2015;17. doi:10.1038/gim.2014.190

5 Park IJ, Yu CS, Kim HC, Jung YH, Han KR, Kim JC. Metachronous colorectal cancer. Colorectal Dis 2006;8. doi:10.1111/j.1463-1318.2006.00949.x

6 Huang W, Yang J, Liu Y, Liu C, Zhang X, Fu W, Shi L, Liu G. Assessing health-related quality of life of patients with colorectal cancer using EQ-5D-5L: a cross-sectional study in Heilongjiang of China. BMJ Open 2018;8:e022711e. http://dx.doi.org/10.1136/bmjopen-2018-022711

7 Engel C, Rahner N, Schulmann K, et al.; German HNPCC Consortium. Efficacy of annual colonoscopic surveillance in individuals with hereditary nonpolyposis colorectal cancer. Clin Gastroenterol Hepatol 2010;8:174–182.
